# Effect of Baricitinib and Adalimumab in Reducing Pain and Improving Function in Patients with Rheumatoid Arthritis in Low Disease Activity: Exploratory Analyses from RA-BEAM

**DOI:** 10.3390/jcm8091394

**Published:** 2019-09-05

**Authors:** Bruno Fautrel, Bruce Kirkham, Janet E. Pope, Tsutomu Takeuchi, Carol Gaich, Amanda Quebe, Baojin Zhu, Inmaculada de la Torre, Francesco De Leonardis, Peter C. Taylor

**Affiliations:** 1 Rheumatology Dept, Institut Pierre Louis d’Epidémiologie et de Santé Publique, Sorbonne Université-Assistance Publique Hôpitaux de Paris, 75013 Paris, France; 2 Department of Rheumatology, Pitié-Salpêtrière Hospital, Assistance Publique-Hôpitaux de Paris, 83 bd de l’hôpital, 75013 Paris, France; 3 Department of Rheumatology, Guys and St Thomas’ NHS Trust, Great Maze Pond, London SE1 9RT, UK; 4 Department of Medicine, Division of Rheumatology, University of Western Ontario, 1151 Richmond St, London, ON N6A 3K7, Canada; 5 Keio University School of Medicine, 35 Shinanomachi, Shinjuku-ku, Tokyo 160-8582, Japan; 6 Eli Lilly and Company, Indianapolis, IN 46285, USA; 7 Botnar Research Centre, NDORMS, University of Oxford, Old Road, Oxford OX3 7LD, UK

**Keywords:** rheumatoid arthritis, baricitinib, pain, recovery of function, fatigue, productivity

## Abstract

Patients with rheumatoid arthritis (RA) may experience residual pain and functional impairment despite good control of disease activity. This study compared improvements in pain and physical function in patients with well-controlled RA after 24 weeks’ treatment with baricitinib, adalimumab or placebo in the 52-week RA-BEAM phase III study. Adults with active RA and inadequate response to methotrexate received baricitinib 4 mg once daily, adalimumab 40 mg every two weeks or placebo, with background methotrexate. Patients (*N* = 1010) were categorised as in remission, in remission or low disease activity, or not in remission or low disease activity at week 24. For patients in remission or low disease activity (*n* = 310), improvements in mean pain and physical function scores at week 24 were significantly greater with baricitinib than placebo (*p* < 0.001 and *p* < 0.01, respectively) and adalimumab (*p* < 0.05 for both). For both outcomes, differences between adalimumab and placebo were not significant. The proportions of patients in remission or low disease activity with minimal or no pain and with normalised physical function were numerically greater with baricitinib than placebo. Baricitinib 4 mg once daily provided enhanced improvement in pain and physical function in patients with well-controlled RA, suggesting it may produce effects beyond immunomodulation.

## 1. Introduction

A major goal in the contemporary treatment of rheumatoid arthritis (RA) is to achieve remission or low disease activity, with the aim of reducing inflammation to prevent joint damage and physical disability [1,2]. However, achieving this objective target is not always associated with a corresponding improvement in disability (Health Assessment Questionnaire (HAQ)) scores and other patient-reported outcomes (PROs): In a study of the Leiden Early Arthritis Clinic cohort, for example, patients with RA were now diagnosed after a shorter duration of symptoms and with less inflammation than they were 20 years ago, but HAQ results remained stable over this time while PROs worsened [3]. Indeed, residual pain and functional impairment can persist despite ongoing treatment and can negatively affect quality of life [4,5]. Control of pain and maintenance of physical function are priorities for patients with RA [5,6,7,8,9]. Improvement in PROs should, therefore, be considered an important treatment goal, in addition to reducing inflammation, for improving the health outcomes of such patients [10].

Baricitinib is an oral selective inhibitor of Janus kinase (JAK)1 and JAK2 [11], which are essential for the intracellular signalling of various cytokines associated with inflammation in RA [12,13]. It is approved for the treatment of moderately to severely active RA in adults in over 50 countries, including the USA, Europe and Japan [14,15,16]. The efficacy and safety of baricitinib as a treatment for RA were established in four phase III, randomised, double-blind, multicentre studies in patients with active disease [17,18,19,20].

The objective of these post-hoc analyses was to compare improvements in pain, physical function, fatigue and work productivity/loss between baricitinib, adalimumab and placebo, all given with background methotrexate, in patients with well-controlled RA (in remission or low disease activity) at week 24 in RA-BEAM.

## 2. Materials and Methods

### 2.1. RA-BEAM Study Design

RA-BEAM (NCT01710358) was a phase III, double-blind, placebo- and active-controlled study in which patients with active RA were randomised to treatment with baricitinib 4 mg, adalimumab or placebo in addition to background methotrexate for 52 weeks (24 weeks for placebo). All patients had an inadequate response to stable doses of methotrexate before study entry. The study design has been described in detail previously [20]. In brief, 1305 adult patients (aged ≥18 years) with moderately to severely active RA were randomised and treated with either baricitinib 4 mg once daily (*N* = 487), adalimumab 40 mg once every two weeks (*N* = 330) or once-daily placebo (*N* = 488) for 52 weeks (24 weeks for placebo) in addition to stable background methotrexate.

RA-BEAM was conducted in accordance with the ethical principles of the 1964 Declaration of Helsinki and its later amendments, and Good Clinical Practice guidelines, and was approved by each centre’s institutional review board or ethics committee. Informed consent was obtained from all individual participants included in the study.

### 2.2. Outcomes Relevant to the Post-Hoc Analyses

Pain was assessed throughout the study, including at week 24, using a pain visual analogue scale (VAS, 0–100 mm), whereas physical function was assessed using the HAQ-Disability Index (HAQ-DI) [21]. Additional PROs assessed at baseline and week 24 included fatigue, measured using the Functional Assessment of Chronic Illness Therapy-Fatigue (FACIT-F) scale [22], and work absenteeism, presenteeism, productivity loss and activity impairment, measured using the Work Productivity and Activity Impairment Questionnaire-RA [23].

### 2.3. Statistical Analysis

Patients from all treatment groups were categorised as being in remission, being in remission or low disease activity, or not being in remission or low disease activity at week 24 (Table 1). Remission was defined as Disease Activity Score for 28-joint count with erythrocyte sedimentation rate (DAS28-ESR) <2.6 and low disease activity as DAS28-ESR ≥2.6 and ≤3.2. Not being in remission or low disease activity was defined as DAS28-ESR >3.2 based on observed data. Changes from baseline to week 24 in pain VAS, HAQ-DI and FACIT-F scores and work-related outcomes were compared between baricitinib, adalimumab and placebo according to remission or low disease activity status. Comparisons were made using analysis of covariance (ANCOVA) adjusted for randomisation variables (region and baseline joint erosion status (1–2 or ≥3)) and baseline score. The proportions of patients achieving minimal or no pain and those achieving normalisation of physical function at week 24 were also compared descriptively between treatment groups according to remission status. Minimal or no pain was defined as a VAS score of ≤10 mm, and normalisation of physical function was defined as a HAQ-DI score of <0.5 (including patients with a baseline HAQ-DI score of <0.5). For all outcome measures, missing values were imputed using modified last observation carried forward (mLOCF) as per the original predefined study analyses [20]. Analyses were not controlled for multiple testing.

For changes from baseline to week 24 in pain and HAQ-DI scores, sensitivity analyses were conducted for patients in remission or low disease activity according to DAS28 with high-sensitivity C-reactive protein (DAS28-hsCRP), Simplified Disease Activity Index (SDAI) or Clinical Disease Activity Index (CDAI) criteria (Table 1).

## 3. Results

Baseline characteristics of 1305 randomised and treated patients in RA-BEAM are shown in Table 2. Characteristics were similar across the treatment groups, including the proportions taking steroids and/or concomitant conventional synthetic disease-modifying antirheumatic drugs. Of these patients, 1010 were included in the current analyses—168 (baricitinib, *n* = 87; adalimumab, *n* = 57; placebo, *n* = 24) were in remission, 310 (baricitinib, *n* = 154; adalimumab, *n =* 110; placebo, *n* = 46) were in remission or low disease activity and 700 (baricitinib, *n* = 267; adalimumab, *n* = 157; placebo, *n* = 276) were not in remission or low disease activity at week 24, according to DAS28-ESR criteria.

### 3.1. Change in Pain VAS Scores

**Figure 1:**
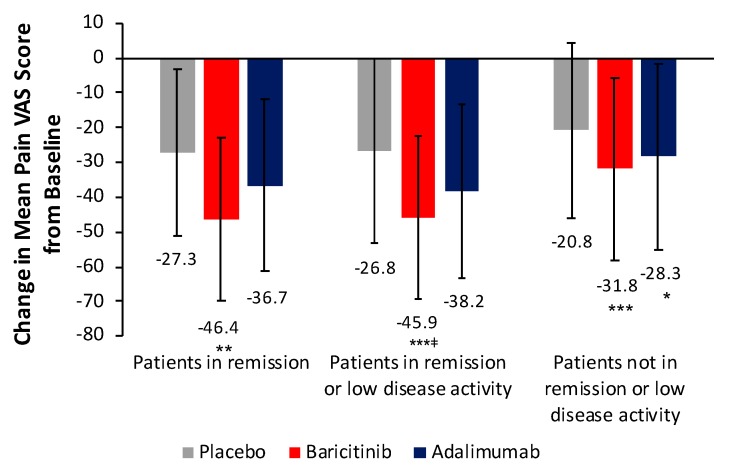
Change from baseline in pain VAS score at week 24 by remission status in patients from RA-BEAM. * *p* < 0.05, ** *p* < 0.01, *** *p* < 0.001 vs. placebo; ^ǂ^ *p* < 0.05 vs. adalimumab. Error bars indicate standard deviation. Change in pain VAS score based on numbers of patients from RA-BEAM in remission (DAS28-ESR <2.6): PBO+MTX *n* = 24, BARI+MTX *n* = 87, ADA+MTX *n* = 57; in remission or low disease activity (DAS28-ESR ≥2.6 and ≤3.2): PBO+MTX *n* = 46, BARI+MTX *n* = 154, ADA+MTX *n* = 110; and not in remission or low disease activity: PBO+MTX *n* = 276, BARI+MTX *n =* 266, ADA+MTX *n* = 157. One patient was missing from the BARI+MTX group for patients not in remission or low disease activity. *ADA* adalimumab, *BARI* baricitinib, *DAS28-ESR* Disease Activity Score for 28-joint count with erythrocyte sedimentation rate, *PBO* placebo, *MTX* methotrexate, *VAS* visual analogue scale.

For patients in remission, change from baseline in mean pain VAS score at week 24 with baricitinib was significantly greater than that with placebo (*p* < 0.01) and greater than that achieved with adalimumab, although the difference between baricitinib and adalimumab was not statistically significant (Figure 1). There was no significant difference between adalimumab and placebo. For patients in remission or low disease activity, change from baseline in mean pain VAS score at week 24 was significantly greater with baricitinib than with placebo (*p* < 0.001) and adalimumab (*p* < 0.05). The difference between adalimumab and placebo was not statistically significant. Results of sensitivity analyses using other disease activity measures were consistent with these findings. For patients not in remission or low disease activity, change from baseline in mean pain VAS score at week 24 was significantly greater with baricitinib and adalimumab than with placebo (*p* < 0.0001 and *p* = 0.0130, respectively). There was no significant difference between baricitinib and adalimumab.

### 3.2. Change in HAQ-DI Scores

**Figure 2:**
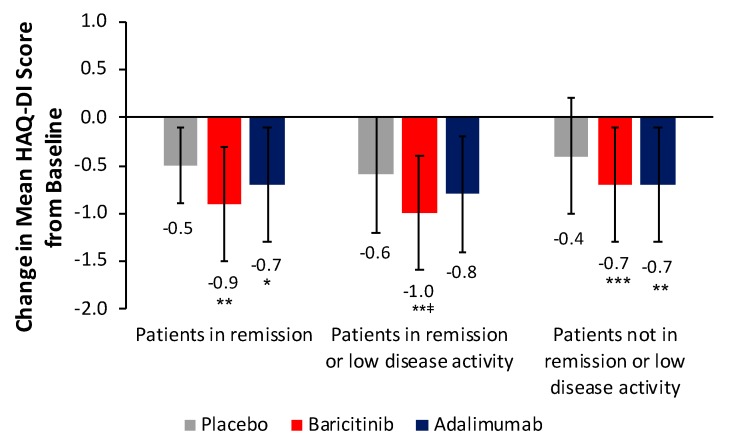
Change from baseline in HAQ-DI score at week 24 by remission status in patients from RA-BEAM. * *p* < 0.05, ** *p* < 0.01, *** *p* < 0.001 vs. placebo; ^ǂ^ *p* < 0.05 vs. adalimumab. Error bars indicate standard deviation. Change in HAQ-DI score based on numbers of patients from RA-BEAM in remission (DAS28-ESR <2.6): PBO+MTX *n* = 24, BARI+MTX *n* = 87, ADA+MTX *n* = 57; in remission or low disease activity (DAS28-ESR ≥2.6 and ≤3.2): PBO+MTX *n* = 46, BARI+MTX *n* = 154, ADA+MTX *n* = 110; and not in remission or low disease activity: PBO+MTX *n* = 276, BARI+MTX *n* = 266, ADA+MTX *n* = 156. One patient was missing from the BARI+MTX group and one from the ADA+MTX group for patients not in remission or low disease activity. *ADA* adalimumab, *BARI* baricitinib, *DAS28-ESR* Disease Activity Score for 28-joint count with erythrocyte sedimentation rate, *HAQ-DI* Health Assessment Questionnaire-Disability Index, *MTX* methotrexate, *PBO* placebo.

For patients in remission, change from baseline in mean HAQ-DI score at week 24 was significantly greater with baricitinib and adalimumab than with placebo (*p* < 0.01 and *p* < 0.05, respectively) (Figure 2). The difference between baricitinib and adalimumab was not statistically significant. For patients in remission or low disease activity, change from baseline in HAQ-DI score at week 24 was significantly greater with baricitinib than with placebo (*p* < 0.01) and adalimumab (*p* < 0.05). There was no significant difference between adalimumab and placebo. Results of sensitivity analyses using other disease activity measures were consistent with these findings (data not shown). For patients not in remission or low disease activity, change from baseline in mean HAQ-DI score at week 24 was significantly greater with baricitinib and adalimumab than with placebo (*p* < 0.0001 and *p* = 0.0014, respectively). The difference between baricitinib and adalimumab was not statistically significant.

### 3.3. Proportion of Patients Achieving Minimal or No Pain and Proportion Achieving Normalisation of Physical Function

**Figure 3:**
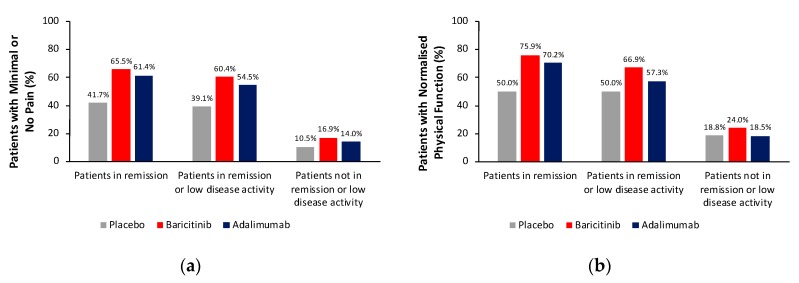
Patients from RA-BEAM with (**a**) minimal/no pain and (**b**) normalised physical function at week 24, by remission status. Proportions of patients from RA-BEAM with (**a**) minimal or no pain (pain VAS ≤10 mm) and (**b**) normalised physical function (HAQ-DI <0.5) based on numbers of patients in remission (DAS28-ESR <2.6): PBO+MTX *n* = 24, BARI+MTX *n* = 87, ADA+MTX *n* = 57; in remission or low disease activity (DAS28-ESR ≥2.6 and ≤3.2): PBO+MTX *n* = 46, BARI+MTX *n* = 154, ADA+MTX *n* = 110; and not in remission or low disease activity (DAS28-ESR >3.2): PBO+MTX *n* = 276, BARI+MTX *n* = 266, ADA+MTX *n* = 156. One patient was missing from the BARI+MTX group and one from the ADA+MTX group for patients not in remission or low disease activity. For the pain analysis, the number of patients not in remission or low disease activity was PBO+MTX *n* = 276, BARI+MTX *n* = 210, ADA+MTX *n* = 120. *ADA* adalimumab, *BARI* baricitinib, *DAS28-ESR* Disease Activity Score for 28-joint count with erythrocyte sedimentation rate, *HAQ-DI* Health Assessment Questionnaire-Disability Index, *MTX* methotrexate, *PBO* placebo, *VAS* visual analogue scale.

The proportion of patients in remission who achieved minimal or no pain at week 24 was 65.5% (57/87) for baricitinib, 61.4% (35/57) for adalimumab and 41.7% (10/24) for placebo (Figure 3a). The proportion of patients in remission who achieved normalised physical function at week 24 was 75.9% (66/87) for baricitinib, 70.2% (40/57) for adalimumab and 50.0% (12/24) for placebo (Figure 3b). Trends were the same, although proportions achieving these endpoints were slightly lower, for patients in remission or low disease activity.

### 3.4. Changes in Other Patient-Reported Outcomes

**Figure 4:**
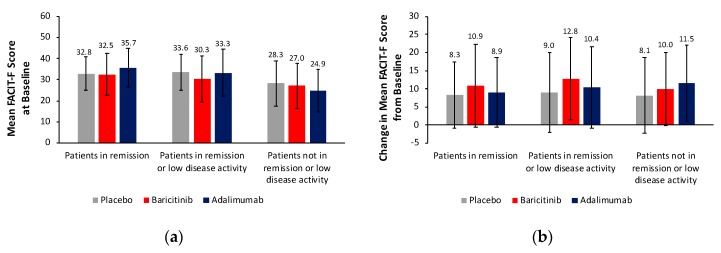
FACIT-F scores in patients from RA-BEAM by remission status (**a**) at baseline and (**b**) change at week 24. Error bars indicate standard deviation. FACIT-F scores at baseline and change in FACIT-F scores based on numbers of patients from RA-BEAM in remission (DAS28-ESR <2.6): PBO+MTX *n* = 24, BARI+MTX *n* = 87, ADA+MTX *n* = 57; in remission or low disease activity (DAS28-ESR ≥2.6 and ≤3.2): PBO+MTX *n* = 46, BARI+MTX *n* = 154, ADA+MTX *n* = 110; and not in remission or low disease activity (DAS28-ESR >3.2): PBO+MTX *n* = 276, BARI+MTX *n* = 266, ADA+MTX *n* = 156. One patient was missing from the BARI+MTX group and one from the ADA+MTX group for patients not in remission or low disease activity. *ADA* adalimumab, *BARI* baricitinib, *DAS28-ESR* Disease Activity Score for 28-joint count with erythrocyte sedimentation rate, *FACIT-F* Functional Assessment of Chronic Illness Therapy-Fatigue, *MTX* methotrexate, *PBO* placebo.

Despite meeting DAS28-ESR criteria for remission or low disease activity, patients continued to experience fatigue, although FACIT-F scores were <36 in all treatment groups (Figure 4). Work productivity of working patients generally improved in all treatment groups, including those not in remission or low disease activity (Supplementary Table S1). For working patients in remission, improvements in work-related measures were numerically greater with both active treatments than with placebo. For those in remission or low disease activity, improvement in activity impairment was significantly greater with baricitinib than placebo, whereas the difference between adalimumab and placebo was not statistically significant. For working patients not in remission or low disease activity, improvements in the proportion of patients present at work, productivity loss and activity impairment were significantly greater with both active treatments than with placebo.

## 4. Discussion

Residual pain and impaired function persist in many patients with RA despite the achievement of disease control [4,5,24]. This residual pain may be non-inflammatory in origin, caused by sensitisation of nociceptors or peripheral joint damage; or it may be due to central sensitisation [25,26,27]. Results of the post-hoc analyses reported here suggest that, in patients with moderately to severely active RA and an inadequate response to methotrexate, addition of baricitinib may be more effective than adalimumab and placebo in improving pain and physical function in patients with a good level of disease control (i.e., in remission or low disease activity). Among patients in remission, significantly greater improvements in pain and physical function were observed with baricitinib than with continued methotrexate alone (placebo group). Among patients in remission or low disease activity, greater improvements in pain and physical function were observed with the addition of baricitinib than with the addition of adalimumab or with continued methotrexate alone. Notably, both active treatments were significantly more effective than placebo at improving pain and physical function in patients who did not achieve controlled disease during the study (not in remission or low disease activity).

Another analysis of data from RA-BEAM also showed that, among patients with varying levels of inflammation, patients treated with baricitinib achieved consistent pain relief regardless of the CRP level at week 24 [28]. Furthermore, patients treated with baricitinib achieved greater and more rapid pain relief than those receiving adalimumab or placebo. Using CRP levels as a surrogate for inflammation, baricitinib plus methotrexate was associated with greater relief from the non-inflammatory component of RA-associated pain than adalimumab plus methotrexate or placebo plus methotrexate [28].

The mechanisms underlying the effect of baricitinib on non-inflammatory pain are not understood. As reviewed by Taylor et al. [28], it is possible that inhibition of JAK1 and JAK2 also produces anti-nociceptive effects that are not related to inflammation, such as inhibition of the JAK2-dependent cytokine granulocyte-macrophage colony-stimulating factor, which may be involved in the pathophysiology of pain [29], and/or inhibition of the JAK2-dependent signal transducer and activator of transcription (STAT)3 phosphorylation pathway [30]. Further studies to elucidate the underlying mechanisms are warranted.

Both greater disease activity and longer disease duration have been shown to increase the likelihood of a patient retiring early or stopping work because of RA [31]. In RA-BEAM, work productivity generally improved in all working patients, including those who did not achieve remission or low disease activity, but not all work-related problems were resolved (Supplementary Table S1). Control of all aspects of the disease, including reduction of pain and fatigue and maintenance of function, is an important goal of RA treatment, and patients generally consider these specified outcomes more essential than control of inflammation [10]. Pain and physical function have been identified as key unmet needs, both clinically and for patients themselves, that can adversely affect a patient’s ability to function normally and their overall well-being [5].

Control of pain in patients with RA is important, since pain has been shown to contribute to worse long-term outcomes. For example, high pain levels at disease onset have been identified as a risk factor for being in the most disabled tertile of RA patients 5–18 years after disease onset [32]. High baseline pain scores were also shown to predict suboptimal mental health (*p* = 0.02) in a cohort of South African patients with early RA [33].

Maintenance of normal physical function is also important in patients with RA. Data from the Dutch DREAM registry showed that having better physical function (measured using the HAQ-DI) was associated with work participation (odds ratio (OR) 0.32, *p* = 0.000) and with starting work after 2 years of treatment with tumour necrosis factor inhibitors (OR 0.58, *p* < 0.1) [34]. Similarly, a survey of patients with RA from France showed a high correlation between deteriorating function and work capacity, such that only 15% of patients with a HAQ score of ≥2 were working, compared with 63% of patients with a HAQ score of <1 [35]. Poor physical function can also affect health-related quality of life [33].

The assessment of PROs, such as pain and function, in RA should help clinicians to focus more on the impact of the disease on patients themselves and how they are feeling rather than solely on the inflammatory component of the disease [10]. This is likely to aid in shared decision-making discussions between patients and clinicians, enabling clinicians to provide more effective and efficient patient care [25]. Indeed, it has been recommended that assessment of the PROs of pain and physical function (HAQ) be added to the current core set of treatment targets to achieve greater patient involvement in the RA treatment process [10]. Our results suggest that patients who do not respond to treatment, as measured using inflammation-associated endpoints, may still experience treatment benefit, and may, therefore, choose to continue with treatment.

Despite major advances in the treatment of RA, predicting remission a priori at the start of disease-modifying treatment remains a clinical conundrum. Nevertheless, in line with recent treatment recommendations for a treat-to-target approach, the aim of any treating rheumatologist should be to help their patients achieve remission or at least low disease activity [1,2]. With respect to this, it could be of clinical relevance to know if differences exist between agents in treating residual symptoms (such as pain) once inflammation is well controlled. Since baricitinib has demonstrated more effective pain control than adalimumab [28], we were interested in investigating whether this benefit persists even when a good level of disease control has been achieved. The post-hoc analyses reported here are not, on their own, intended to inform clinical practice, but to help towards better defining current evidence for the pain-relieving benefits of baricitinib compared to tumour necrosis factor inhibitors.

Limitations of the current analysis include that post-hoc analyses are exploratory by nature, aimed at creating hypotheses rather than clearly demonstrable facts; the sample sizes for patients achieving remission or low disease activity and working patients were small; and generalisability of the results to patients in routine care who receive baricitinib or adalimumab is uncertain. In addition, analyses were not adjusted for multiple testing.

## 5. Conclusions

Treatment with baricitinib 4 mg once daily or adalimumab 40 mg every other week resulted in improvements in pain, physical function, fatigue and work productivity/impairment in patients with RA, independent of the impact on inflammation, measured using DAS28-ESR. Among patients in remission or low disease activity, greater improvements in pain and physical function were observed with baricitinib than with adalimumab and placebo. Research to better understand the role of JAK1/JAK2 pathways in the control of pain beyond the regulation of inflammation is underway to help clarify the differential effects of baricitinib relative to adalimumab on pain.

## Figures and Tables

**Table 1:** Remission and remission or low disease activity rates at week 24 in patients with moderately to severely active rheumatoid arthritis from RA-BEAM [14,20].

Treatment ^¥^	BARI 4 mg	ADA 40 mg Q2W	Placebo
*n*	487	330	488
**Remission rates (%)**			
DAS28-ESR <2.6	18 ***	18 ***	5
DAS28-hsCRP <2.6	34 ***	32 ***	8
SDAI ≤3.3	16 ***	14 ***	3
CDAI ≤2.8	16 ***	12 ***	4
**Remission or low disease activity rates (%)**			
DAS28-ESR ≤3.2	32 ***	34 ***	10
DAS28-hsCRP ≤3.2	52 ***	48 ***	19
DAS28-ESR ≥2.6 and ≤3.2	14	16	5
DAS28-hsCRP ≥2.6 and ≤3.2	18	16	11
SDAI >3.3 and ≤11	35	34	17
CDAI >2.8 and ≤10	34	36	16

^¥^ Patients remained on background methotrexate throughout the study; all patients were bDMARD naïve. *** *p* < 0.001 vs. placebo. ADA adalimumab, BARI baricitinib, CDAI Clinical Disease Activity Index, bDMARD biologic disease-modifying antirheumatic drug, DAS28-ESR Disease Activity Score for 28-joint count with erythrocyte sedimentation rate, DAS28-hsCRP Disease Activity Score for 28-joint count with high-sensitivity C-reactive protein, Q2W once every two weeks, SDAI Simplified Disease Activity Index.

**Table 2:** Baseline characteristics of 1305 randomised and treated patients in RA-BEAM, and the 168 patients in remission (DAS28-ESR <2.6) at week 24 [20].

Characteristic	All Randomised and Treated Patients (*N* = 1305)			Patients in Remission at Week 24 (*N* = 168)		
	Placebo (*N* = 488)	Baricitinib 4 mg (*N* = 487)	Adalimumab (*N* = 330)	Placebo (*N* = 24)	Baricitinib 4 mg (*N* = 87)	Adalimumab (*N* = 57)
Age (years)	53 ± 12	54 ± 12	53 ± 12	52 ± 12	52 ± 13	53 ± 13
Female	382 (78)	375 (77)	251 (76)	15 (63)	62 (71)	41 (72)
Time from symptom onset (years)	10.4 ± 9	10.3 ± 9	9.6 ± 9	9.1 ± 6	8.9 ± 9	8.1 ± 8
ACPA positive	424 (87)	427 (88)	295 (89)	22 (92)	75 (86)	52 (91)
RF positive	451 (92)	439 (90)	301 (91)	22 (92)	76 (87)	49 (86)
≥3 erosions	371 (76)	371 (76)	245 (75)	17 (71)	66 (77)	41 (75)
mTSS total score	45 ± 50	43 ± 50	44 ± 51	36 ± 41	39 ± 43	37 ± 42
Erosion score	26.8 ± 29	25.1 ± 28	26.4 ± 29	22.5 ± 24	24.4 ± 26	22.7 ± 23
Joint space narrowing score	18.2 ± 23	17.3 ± 23	18.0 ± 24	13.1 ± 18	14.6 ± 19	14.7 ± 20
Concomitant corticosteroid use	290 (59)	275 (56)	201 (61)	13 (54)	50 (58)	39 (68)
Type of csDMARD currently used						
MTX only	398 (82)	413 (85)	277 (84)	20 (83)	71 (82)	49 (86)
MTX + other csDMARD	89 (18)	74 (15)	53 (16)	4 (17)	16 (18)	8 (14)
MTX weekly dose in mg	15 ± 5	15 ± 5	15 ± 4	15 ± 4	14 ± 5	14 ± 5
DAS28-hsCRP	5.7 ± 1.0	5.8 ± 0.9	5.8 ± 0.9	5.0 ± 0.8	5.3 ± 1.0	5.2 ± 0.9
DAS28-ESR	6.4 ± 1.0	6.5 ± 0.9	6.4 ± 1.0	5.4 ± 0.8	5.9 ± 1.0	5.8 ± 0.9
CDAI score	38 ± 13	38 ± 12	38 ± 13	30 ± 10	33 ± 12	32 ± 12
Pain VAS score (0–100 mm) ^¥^	60 ± 23	62 ± 22	61 ± 23	44 ± 21	57 ± 23	48 ± 23
HAQ-DI ^¶^	1.6 ± 0.7	1.6 ± 0.7	1.6 ± 0.7	1.1 ± 0.5	1.3 ± 0.6	1.1 ± 0.7
FACIT-F ^$^	28.6 ± 10.7	28.1 ± 10.7	27.6 ± 11.4	32.8 ± 7.9	32.5 ± 10.0	35.7 ± 9.1

Data are presented as mean ± standard deviation or *n* (%). ^¥^ Higher scores indicate more severe pain. ^¶^ Score range 0–3, with higher scores indicating greater disability [21]. ^$^ Score range 0–52, with lower scores indicating greater fatigue [22]. ACPA anti-citrullinated protein antibody (positivity >10 units/mL), CDAI Clinical Disease Activity Index, csDMARD conventional synthetic disease-modifying antirheumatic drug, DAS28-hsCRP Disease Activity Score in 28 joints using the high-sensitivity C-reactive protein level, DAS28-ESR Disease Activity Score for 28-joint count with erythrocyte sedimentation rate, FACIT-F Functional Assessment of Chronic Illness Therapy-Fatigue, HAQ-DI Health Assessment Questionnaire-Disability Index, mTSS van der Heijde modified total Sharp score, MTX methotrexate, RF rheumatoid factor (positivity >14 units/mL), VAS visual analogue scale.

## Supplementary Materials

The following are available online at https://www.mdpi.com/2077-0383/8/9/1394/s1, Table S1: Work-related patient-reported outcomes at week 24 according to remission status in working patients from RA-BEAM.

## References

[1] Singh J.A., Saag K.G., Bridges S.L., Akl E.A., Bannuru R.R., Sullivan M.C., Vaysbrot E., McNaughton C., Osani M., Shmerling R.H. (2016). 2015 American College of Rheumatology guideline for the treatment of rheumatoid arthritis. Arthritis Rheumatol..

[2] Smolen J.S., Landewé R., Bijlsma J., Burmester G., Chatzidionysiou K., Dougados M., Nam J., Ramiro S., Voshaar M., Van Vollenhoven R. (2017). EULAR recommendations for the management of rheumatoid arthritis with synthetic and biological disease-modifying antirheumatic drugs: 2016 update. Ann. Rheum. Dis..

[3] Nieuwenhuis W.P., De Wit M.P., Boonen A., Van Der Helm-Van Mil A.H. (2016). Changes in the clinical presentation of patients with rheumatoid arthritis from the early 1990s to the years 2010: Earlier identification but more severe patient reported outcomes. Ann. Rheum. Dis..

[4] Ishiguro N., Dougados M., Cai Z., Zhu B., Ishida M., Sato M., Gaich C., Quebe A., Stoykov I., Tanaka Y. (2018). Relationship between disease activity and patient-reported outcomes in rheumatoid arthritis: Post hoc analyses of overall and Japanese results from two phase 3 clinical trials. Mod. Rheumatol..

[5] Taylor P.C., Moore A., Vasilescu R., Alvir J., Tarallo M. (2016). A structured literature review of the burden of illness and unmet needs in patients with rheumatoid arthritis: A current perspective. Rheumatol. Int..

[6] Gossec L., Dougados M., Rincheval N., Balanescu A., Boumpas D.T., Canadelo S., Carmona L., Daurès J.P., de Wit M., Dijkmans B.A. (2009). Elaboration of the preliminary Rheumatoid Arthritis Impact of Disease (RAID) score: A EULAR initiative. Ann. Rheum. Dis..

[7] Khan N.A., Spencer H.J., Abda E., Aggarwal A., Alten R., Ancuta C., Andersone D., Bergman M., Craig-Muller J., Detert J. (2012). Determinants of discordance in patients’ and physicians’ rating of rheumatoid arthritis disease activity. Arthritis Care Res..

[8] Wen H., Ralph Schumacher H., Li X., Gu J., Ma L., Wei H., Yokogawa N., Shiroto K., Baker J.F., Dinnella J. (2012). Comparison of expectations of physicians and patients with rheumatoid arthritis for rheumatology clinic visits: A pilot, multicenter, international study. Int. J. Rheum. Dis..

[9] Van Tuyl L.H., Sadlonova M., Hewlett S., Davis B., Flurey C., Goel N., Gossec L., Brahe C.H., Hill C.L., Hoogland W. (2017). The patient perspective on absence of disease activity in rheumatoid arthritis: A survey to identify key domains of patient-perceived remission. Ann. Rheum. Dis..

[10] Fautrel B., Alten R., Kirkham B., De La Torre I., Durand F., Barry J., Holzkaemper T., Fakhouri WTaylor P.C. (2018). Call for action: How to improve use of patient-reported outcomes to guide clinical decision making in rheumatoid arthritis. Rheumatol. Int..

[11] Fridman J.S., Scherle P.A., Collins R., Burn T.C., Li Y., Li J., Covington M.B., Thomas B., Collier P., Favata M.F. (2010). Selective inhibition of JAK1 and JAK2 is efficacious in rodent models of arthritis: Preclinical characterization of INCB028050. J. Immunol..

[12] O’Shea J.J., Kontzias A., Yamaoka K., Tanaka Y., Laurence A. (2013). Janus kinase inhibitors in autoimmune diseases. Ann. Rheum. Dis..

[13] Choy E.H.S., Miceli-Richard C., González-Gay M.A., Sinigaglia L., Schlichting D.E., Meszaros G., de la Torre I., Schulze-Koops H. (2019). The effect of JAK/JAK2 inhibition in rheumatoid arthritis: Efficacy and safety of baricitinib. RMD Open.

[14] European Medicines Agency (2018). Olumiant 2 mg and 4 mg Film-Coated Tablets. http://www.ema.europa.eu/docs/en_GB/document_library/EPAR_-_Product_Information/human/004085/WC500223723.pdf.

[15] Food and Drug Administration (2018). Olumiant (Baricitinib) Tablets, for Oral Use. https://www.accessdata.fda.gov/drugsatfda_docs/label/2018/207924s000lbl.pdf.

[16] Pharmaceutical and Medical Devices Agency (2017). Report on the Deliberation Results. Olumiant Tablets 2 mg, Olumiant Tablets 4 mg. http://www.pmda.go.jp/files/000226301.pdf.

[17] Genovese M.C., Kremer J., Zamani O., Ludivico C., Krogulec M., Xie L., Beattie S.D., Koch A.E., Cardillo T.E., Rooney T.P. (2016). Baricitinib in patients with refractory rheumatoid arthritis. N. Engl. J. Med..

[18] Dougados M., van der Heijde D., Chen Y.C., Greenwald M., Drescher E., Liu J., Beattie S., Witt S., de la Torre I., Gaich CRooney T. (2017). Baricitinib in patients with inadequate response or intolerance to conventional synthetic DMARDs: Results from the RA-BUILD study. Ann. Rheum. Dis..

[19] Fleischmann R., Schiff M., van der Heijde D., Ramos-Remus C., Spindler A., Stanislav M., Zerbini C.A., Gurbuz S., Dickson C., de Bono S. (2017). Baricitinib, methotrexate, or combination in patients with rheumatoid arthritis and no or limited prior disease-modifying antirheumatic drug treatment. Arthritis Rheumatol..

[20] Taylor P.C., Keystone E.C., van der Heijde D., Weinblatt M.E., del Carmen Morales L., Reyes Gonzaga J., Yakushin S., Ishii T., Emoto K., Beattie S. (2017). Baricitinib versus placebo or adalimumab in rheumatoid arthritis. N. Engl. J. Med..

[21] Fries J.F., Spitz P.W., Young D.Y. (1982). The dimensions of health outcomes: The health assessment questionnaire, disability and pain scales. J. Rheumatol..

[22] Cella D., Yount S., Sorensen M., Chartash E., Sengupta N., Grober J. (2005). Validation of the Functional Assessment of Chronic Illness Therapy Fatigue Scale relative to other instrumentation in patients with rheumatoid arthritis. J. Rheumatol..

[23] Reilly M.C., Zbrozek A.S., Dukes E.M. (1993). The validity and reproducibility of a work productivity and activity impairment instrument. Pharmacoeconomics.

[24] Ishida M., Kuroiwa Y., Yoshida E., Sato M., Krupa D., Henry N., Ikeda K., Kaneko Y. (2018). Residual symptoms and disease burden among patients with rheumatoid arthritis in remission or low disease activity: A systematic literature review. Mod. Rheumatol..

[25] Taylor P., Manger B., Alvaro-Gracia J., Johnstone R., Gomez-Reino J., Eberhardt E., Wolfe F., Schwartzman S., Furfaro N., Kavanaugh A. (2010). Patient perceptions concerning pain management in the treatment of rheumatoid arthritis. J. Int. Med. Res..

[26] Walsh D.A., Mcwilliams D.F. (2014). Mechanisms, impact and management of pain in rheumatoid arthritis. Nat. Rev. Rheumatol..

[27] Boyden S.D., Hossain I.N., Wohlfahrt A., Lee Y.C. (2016). Non-inflammatory causes of pain in patients with rheumatoid arthritis. Curr. Rheumatol. Rep..

[28] Taylor P.C., Lee Y.C., Fleischmann R., Takeuchi T., Perkins E.L., Fautrel B., Zhu B., Quebe A.K., Gaich C.L., Zhang X. (2019). Achieving pain control in rheumatoid arthritis with baricitinib or adalimumab plus methotrexate: Results from the RA-BEAM trial. J. Clin. Med..

[29] Cook A.D., Pobjoy J., Steidl S., Dürr M., Braine E.L., Turner A.L., Lacey D.C., Hamilton J.A. (2012). Granulocyte-macrophage colony-stimulating factor is a key mediator in experimental osteoarthritis pain and disease development. Arthritis Res. Ther..

[30] Dominguez E., Rivat C., Pommier B., Mauborgne A., Pohl M. (2008). JAK/STAT3 pathway is activated in spinal cord microglia after peripheral nerve injury and contributes to neuropathic pain development in rat. J. Neurochem..

[31] Capron J., De Leonardis F., Fakhouri W., Burke T., Rose A., Jacob I. (2017). The impact of rheumatoid arthritis (RA) on a patient’s ability to stay in work and level of pain experienced. Value Health.

[32] Malm K., Bergman S., Andersson M., Bremander A., BARFOT Study Group (2015). Predictors of severe self-reported disability in RA in a long-term follow-up study. Disabil. Rehabil..

[33] Hodkinson B., Musenge E., Ally M., Meyer P.W., Anderson R., Tikly M. (2012). Functional disability and health-related quality of life in South Africans with early rheumatoid arthritis. Scand. J. Rheumatol..

[34] Manders S.H., Kievit W., Braakman-Jansen A.L., Brus H.L., Hendriks L., Fransen J., van de Laar M.A., van Riel P.L. (2014). Determinants associated with work participation in patients with established rheumatoid arthritis taking tumor necrosis factor inhibitors. J. Rheumatol..

[35] Kobelt G., Woronoff A.S., Richard B., Peeters P., Sany J. (2008). Disease status, costs and quality of life of patients with rheumatoid arthritis in France: The ECO-PR study. Jt. Bone Spine.

