# Supplementary material for: Effect of Baricitinib and Adalimumab in Reducing Pain and Improving Function in Patients with Rheumatoid Arthritis in Low Disease Activity: Exploratory Analyses from RA-BEAM
Source: J Clin Med. 2019 Sep 5;8(9):1394. doi: 10.3390/jcm8091394 (PMC6780319; doi:10.3390/jcm8091394)
Supplement: Supplementary file 1 [file jcm-08-01394-s001.pdf]

Journal of Clinical Medicine

MDPI

Supplementary Table S1. Work-related patient-reported outcomes at week 24 according to remission status in working patients from RA-BEAM.

PRO Measure	n	Baseline	Mean Value at Week 24	Observed Change at week 24	n	Baseline	Mean Value at Week 24	Observed Change at Week 24	n	Baseline	Mean Value at Week 24	Observed Change at Week 24
	**Patients in remission on placebo (n = 24)**				**Patients in remission on baricitinib 4 mg (n = 87)**				**Patients in remission on adalimumab (n = 57)**			
Absenteeism (%)	10	7.1	4.3	−2.8	34	5.2	5.9	0.7	34	4.1	1.5	−2.5
Present at work (%)	10	22.0	11.0	−11.0	34	45.9	9.7	−36.2	34	33.5	11.5	−22.1
Productivity loss (%)	10	24.8	13.1	−11.7	34	47.7	14.1	−33.6	34	35.3	13.0	−22.3
Activity impairment (%)	24	37.5	17.5	−20.0	87	52.1	14.4	−37.7	57	41.2	14.6	−26.7
	**Patients in remission or low disease activity on placebo (n = 46)**				**Patients in remission or low disease activity on baricitinib 4 mg (n = 154)**				**Patients in remission or low disease activity on adalimumab (n = 110)**			
Absenteeism (%)	21	3.5	4.4	0.9	66	5.9	6.8	0.9	62	5.9	5.3	−0.5
Present at work (%)	21	33.8	16.2	−17.6	66	43.0	13.2	−29.8	61	37.4	13.8	−23.6
Productivity loss (%)	21	35.2	19.1	−16.1	66	45.6	18.7	−26.9	61	39.7	15.9	−23.8
Activity impairment (%)	46	43.5	20.0	−23.5	154	52.7	15.9	**−36.8***	110	47.3	17.0	−30.3
	**Patients not in remission or low disease activity on placebo (n = 276)**				**Patients not in remission or low disease activity on baricitinib 4 mg (n = 267) ¥**				**Patients not in remission or low disease activity on adalimumab (n = 157) ¶**			
Absenteeism (%)	111	14.5	11.0	−3.5	94	15.0	9.2	−5.8	48	19.5	10.2	−9.3
Present at work (%)	102	45.4	37.2	−8.2	87	45.5	31.6	**−13.9 ***	44	54.8	35.9	**−18.9 ***
Productivity loss (%)	102	48.3	41.3	−7.0	87	48.9	34.6	**−14.3 ****	44	58.2	39.8	**−18.4 ***
Activity impairment (%)	276	57.6	42.7	−15.0	266	60.3	36.4	**−23.9 *****	156	63.7	40.8	**−22.8 ***

At baseline, 41–43% of patients from RA-BEAM reported being employed. Remission was defined as DAS28-ESR <2.6 and low disease activity as DAS28-ESR ≥2.6 and ≤3.2. Numbers in bold are significantly different compared with placebo. *BARI* baricitinib, *DAS28-ESR* Disease Activity Score for 28-joint count with erythrocyte sedimentation rate, *MTX* methotrexate, *PRO* patient-reported outcome. ¥ One patient was missing from the BARI+MTX group. ¶ One patient was missing from the ADA+MTX group. * $p < 0.05$, ** $p < 0.01$, *** $p < 0.001$ vs. place.
